# Antioxidant and Anti-Inflammatory Effect of the Consumption of Powdered Concentrate of *Sechium edule* var. *nigrum spinosum* in Mexican Older Adults with Metabolic Syndrome

**DOI:** 10.3390/antiox11061076

**Published:** 2022-05-28

**Authors:** Taide Laurita Arista-Ugalde, Edelmiro Santiago-Osorio, Alberto Monroy-García, Juana Rosado-Pérez, Itzen Aguiñiga-Sánchez, Jorge Cadena-Iñiguez, Graciela Gavia-García, Víctor Manuel Mendoza-Núñez

**Affiliations:** 1 Research Unit on Gerontology, FES Zaragoza, National Autonomous University of Mexico, Mexico City 09230, Mexico; tdlarista@comunidad.unam.mx (T.L.A.-U.); juanarosadoperez@comunidad.unam.mx (J.R.-P.); ggg1501@hotmail.com (G.G.-G.); 2 Hematopoiesis and Leukemia Laboratory, Research Unit on Cell Differentiation and Cancer, FES Zaragoza, National Autonomous University of Mexico, Mexico City 09230, Mexico; edelmiro@unam.mx; 3 Immunology and Cancer Laboratory, Medical Research Unit in Oncological Diseases, CMN SXXI, Instituto Mexicano del Seguro Social, Mexico City 06720, Mexico; albertomong13@comunidad.unam.mx; 4 Postgraduate College, Campus San Luis Potosí, Iturbide No. 73 Street, Salinas de Hidalgo, San Luis Potosí 78600, Mexico; liberitzen@comunidad.unam.mx; 5 Department of Biomedical Sciences, School of Medicine, FES Zaragoza, National Autonomous University of Mexico, Mexico City 09230, Mexico; jocadena@colpos.mx

**Keywords:** metabolic syndrome, oxidative stress, anti-inflammatory, *Sechium edule*, older adults

## Abstract

Metabolic syndrome (MetS) has a high prevalence in older adults and is a risk factor for cardiovascular diseases and complications of old age. It has also been related to oxidative stress (OxS) and chronic inflammation (CI) and their consequent alterations. Therefore, it is important to propose therapeutic alternatives such as the consumption of *Sechium edule* (Chayote), since hypoglycemic, hypotensive, and lipogenesis inhibitor properties are attributed to it. We carried out a study in 81 older adults (OA) with MetS to determine the effect of consumption of chayote powder concentrate (500 mg, three times a day) for six months, with a baseline measurement, at three and six months in an experimental group (EG) (*n* = 41) and a placebo group (PG) (*n* = 40), all with a diagnosis of MetS according to the criteria of National Adult Treatment Panel of the National Cholesterol Program III (NCEP/ATP III). Anthropometric, biochemical, OxS markers, and inflammation measurements were performed on all participants, basal, three, and six months after. A statistically significant decrease was found in the concentration of lipoperoxides (TBARS), 8-isoprostanes, 8-OHdG, oxidative stress score (OSS), HbA1c, blood pressure, and in the number of MetS diagnostic criteria, as well as an increase in total antioxidant status (TAS), antioxidant gap (GAP), superoxide dismutase (SOD), interleukin 10 (IL-10), and HDL-cholesterol in EG. The results suggest that the consumption of *Sechium edule* powder has a hypotensive, hypoglycemic, antioxidant, and anti-inflammatory effect in OA with MetS and reduced the percentage of patients with MetS.

## 1. Introduction

Oxidative stress (OxS) is a biochemical alteration characterized by an imbalance between the generation of free radicals and antioxidants, in favor of the former, which causes oxidative damage to macromolecules (proteins, carbohydrates, lipids, and DNA). This alteration has been linked to aging and the pathophysiology of more than 100 chronic non-communicable diseases, including metabolic syndrome (MetS) [1,2,3].

On the other hand, it has been pointed out that aging per se occurs with a chronic inflammatory process (CIP) called “inflamm-aging”, which increases vulnerability to the presence of chronic diseases [4]. This inflammatory process is directly linked to OxS and aging, collectively known as “oxi-inflamm-aging” [5]. The biochemical alteration resulting from these processes constitutes a risk factor for several chronic diseases related to aging.

One of the most prevalent health problems in old age is MetS or insulin resistance, which is characterized by the association of several alterations linked pathophysiologically by insulin resistance and hyperinsulinemia. The components of MetS are central or visceral obesity, atherogenic dyslipidemia, arterial hypertension, and insulin resistance with or without glucose intolerance, in a pro-inflammatory state and a pro-thrombotic state. Therefore, MetS is diagnosed when abnormalities such as obesity, hypertriglyceridemia, increased blood pressure, hyperglycemia, and decreased HDL cholesterol are identified. The treatment includes the consumption of several drugs for long periods, giving rise to polypharmacy and its side effects [6].

Therefore, it is necessary to have alternatives that help in the prevention of and reduction in symptoms of this syndrome. In this context, *Sechium edule* (chayote) (*S. edule*) is an edible plant of the *Cucurbitaceae* family, whose phytochemical analysis has shown the presence of cucurbitacins, polyphenols, and flavonoids that give it antioxidant, anti-inflammatory, hypoglycemic, hypotensive, as well as inhibitory effects of lipogenesis [7,8,9,10,11,12].

In a previous exploratory study by our research group on MetS patients, it was shown that the consumption of 1.5 g of *S. edule* powder for six weeks induces a statistically significant decrease in the concentration of lipoperoxides, TNFα, and a decrease in the oxidative stress score (OSS), as well as an increase in total antioxidant status (TAS) [13]. Likewise, in another study, the consumption of *S. edule* for 12 weeks confirmed the findings of the exploratory study. In addition, there was an increase in the enzyme superoxide dismutase (SOD) [14]. However, the results are not conclusive considering the limitations of the sample size and treatment time. For this reason, the purpose of the present study was to consolidate the findings by expanding the sample size and treatment time according to the guidelines of a solid clinical study.

## 2. Materials and Methods

### 2.1. Population and Study Design

Prior to informed consent, a quasi-experimental study approved by the Research Bioethics and Biosafety Committee of the Faculty of Higher Studies Zaragoza, UNAM (23/02-SO/2.4.2) and registered in: ISRCTN43215432 was carried out. The study population was recruited under an open call distributed by social networks where the objectives of the study and the inclusion criteria were specified, such as age over 60 years with obesity or overweight who lived in Mexico City. 

**Figure 1:**
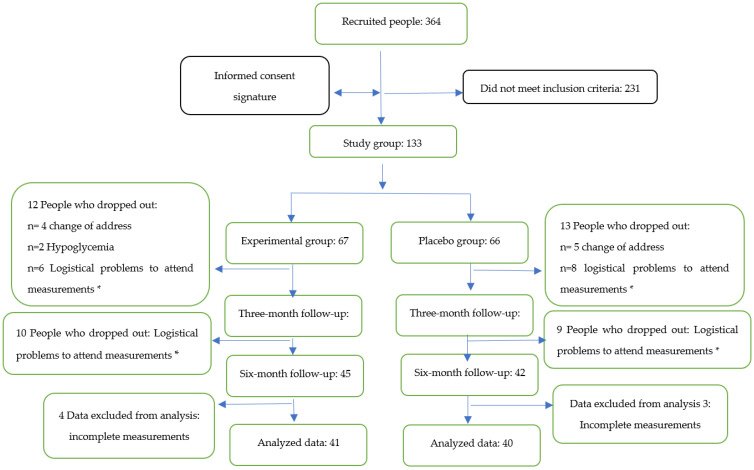
A general outline of the study. * Logistic problems: refers to medical appointments at a different location on the day of the measurement, lack of a companion, long distances to travel, and not enough time available to travel to the location.

A convenience sample of *n* = 133 adults over 60 years of age who met the inclusion criteria was studied, and they were randomly assigned into two groups: (i) experimental (EG) *n* = 67, who consumed three 500 mg capsules of *Sechium edule* per day for six months; (ii) placebo (PG) *n* = 66, who consumed three identical-appearing capsules for the same time period, but without *Sechium edule*. However, during the treatment period, 22 people from the EG and 24 from the PG dropped out of the study due to logistical issues and due to a change of address. For this reason, data from *n* = 41 in the EG and *n* = 40 in the PG were analyzed (Figure 1). 

Measurements of all participants were taken before and after three and six months of treatment: anthropometric and biochemical parameters, serum inflammatory cytokines, plasma lipoperoxides (LPO) and 8-isoprostanes, total antioxidant status (TAS) in plasma, erythrocyte SOD activity, and glutathione peroxidase (GPx) and 8-OHdG concentration.

### 2.2. Diagnosis of Metabolic Syndrome

All participants were diagnosed with MetS according to the National Adult Treatment Panel of the National Cholesterol Program III (NCEP/ATP III): (i) waist circumference > 102 for men or >88 cm for women; (ii) triglycerides ≥ 150 mg/dL; (iii) high-density lipoprotein cholesterol (HDL-C) < 40 mg/dL in men or <50 mg/dL in women; (iv) blood pressure ≥ 130/85 mmHg; and (v) glucose ≥ 110 mg/dL. The presence of three or more of the five parameters was considered a diagnosis of MetS [15]. 

### 2.3. Anthropometric Measures

To appreciate the corporal dimensions, height and weight were determined. Subjects were weighed using a medical scale (Torino), wearing a clinical gown. For height determination, a wall stadiometer was used (SECA, Hamburg, Germany). To obtain a proper determination, subjects were required to step with heels together and heads in contact with the stadiometer. Subjects kept their head upright on Frankfort’s plane, meaning that a horizontal imaginary line was drawn from the ear to the eye’s orbital area. 

To determine the abdominal fat distribution of the subjects, the abdominal circumference was measured at the level of the navel without exerting pressure on the body using an asbestos measuring tape. All determinations were collected by trained personnel of the FES-Z [16].

For the estimation of fat mass (FM) and skeletal muscle mass index (SMMI), bioelectrical impedance analysis (BIA) was performed using four-pole mono-frequency equipment (50 kHz, Quantum X, RJL System). Before the BIA, the participants removed metal objects from their body (jewelry), and the standard technique [17] was applied, placing electrodes on the back of the right hand and foot, thus obtaining the resistance and reactance. With these data, we estimated skeletal muscle mass (SMM), which was calculated using the Janssen equation [18]:

SMM (kg): [(height cm^2^/resistance) × 0.401] + (sex × 3.825) + (age × −0.071) + 5.102.

For gender, males = 1 and females = 0, and age is measured in years.

Finally, to obtain SMMI, the SMM was divided by height squared (m^2^).

SMI = (SMM/Height m^2^)

For fat free mass (FLM), the following equation was used:

FLM (kg) = [(0.7374) × (height cm^2^/resistance)] + [(0.1763 × weight)] − [ (0.1773 × age)] + [ (0.1198 × (reactance) ] − 2.4658

Once we had FLM, we subtracted it from the weight of each patient and obtained the fat mass of each one:

FM (kg) = weight − FLM

### 2.4. Blood Pressure (BP)

BP was measured following the standardized protocol according to the Official Mexican Standard (NOM-030-SSA-1999). Using a mercury manometer in both arms, BP was recorded. Measurements were taken in the morning under fasting condition or two hours after breakfast. Subjects with probable hypertension were identified using the Osler technique. Measurements were taken by trained physicians to standardize the procedure [19].

### 2.5. Biochemical Analysis

Blood samples were collected by venipuncture after an 8 h fast and then placed in vacutainer/siliconized test tubes without anticoagulant for biochemical determinations (glucose, uric acid, lipid profile, cytokines, and 8-OHdG) with ethylenediamine tetraacetic acid (EDTA) as anticoagulant for glycosylated hemoglobin and with heparin for the oxidative stress OxS tests. These were fractioned as follows: 600 µL of whole blood for SOD, 100 µL for GPx, heparinized plasma 100 µL for TAS, and 1000 µL for lipid peroxidation (LPO) were separated. The techniques for SOD, TAS, and GPx were performed at the microscale in multi-well plates, which were read on a Multiskan Go from Thermo Scientific, Denver, CO, USA).

Glucose, uric acid, and lipid profile were determined using colorimetric techniques with an automated Selectra Junior clinical chemistry analyzer (Vital Scientific, Dieren, The Netherlands). For all determinations, the intraassay and interassay variation coefficients were less than 5%. An immunoturbidimetric assay was used for the measurement of glycosylated hemoglobin and serum C reactive protein (CRP) in the same chemistry analyzer.

### 2.6. Plasma Thiobarbituric Acid-Reactive Substances (TBARS)

A thiobarbituric acid-reacting substances (TBARS) assay was used as described in Jentzsch et al. [20]. In this assay, one molecule of malondialdehyde reacts with two molecules of thiobarbituric acid (TBA) to produce a pink pigment with absorption at 535 nm. Amplification of peroxidation during the assay is prevented by the addition of the chain-breaking antioxidant butylated hydroxytoluene. Malondialdehyde standard (0.2–4 µmol/L) was prepared either by hydrolysis of 1,1,3,3-tetramethoxypropane (Sigma-Aldrich Co., St Louis, MO, USA), for the samples (heparinized plasma, in 200 µL), mixed with 200 µL of orthophosphoric acid (0.2 mol/L; Sigma-Aldrich Co., St Louis, MO, USA) and 25 µL of butylated hydroxytoluene (2 mmol/L; Sigma-Aldrich Co., St Louis, MO, USA); then, 25 µL of TBA (0.11 mol/L in 0.1 mol/L NaOH) (Fluka Chemie GmbH, Buchs, Switzerland) was added and mixed. The contents were incubated at 90 °C for 45 min in a water bath. Previously cooled (on ice to prevent further reaction) TBARS were extracted once with 500 µL of *n*-butanol (Sigma-Aldrich Co. St Louis, MO, USA) and were added alongside 50 µL of a saturated solution of NaCl (Sigma, St Louis, MO, USA). The upper butanol phase was read at 535 nm and 572 nm to correct for baseline absorption using a spectrophotometer Multiskan Go (Thermo Scientific, Denver, CO, USA). Malondialdehyde equivalents (TBARS) quantification was performed with the calibration curve.

### 2.7. Total Antioxidant Status in Plasma (TAS)

Plasma total antioxidant status (TAS) levels were quantified using 2,2′-azino-bis (3 ethylbenzthiazoline-6-sulfonic acid) (ABTS) (Randox Laboratories Ltd., Antrim, UK), which is incubated with a peroxidase to produce the radical cation ABTS+. The bluish green staining of the ABTS+ cation is relatively stable and measured at 600 nm; antioxidants present in the plasma cause suppression of this color production to a degree that is proportional to the concentration. The kinetics reaction was measured with a spectrophotometer Multiskan Go Microplate (Thermo Scientific, Denver, CO, USA).

### 2.8. Superoxide Dismutase (SOD)

In this method, superoxide radicals are generated by employing xanthine and xanthine oxidase. The formed radical reacts with 2-(4-iodophenyl)-3-(4-nitrophenol)-5-phenyltetrazolium chloride to form a red formazan color, which is measured at 505 nm. The superoxide dismutase (SOD) in the sample causes the inhibition of this reaction; the SOD activity is proportional to the degree of inhibition of the reaction (Randox Laboratories Ltd., Antrim, UK). Kinetics were measured with a spectrophotometer Multiskan Go (Thermo Scientific, Denver, CO, USA).

### 2.9. Red Blood Cell Glutathione Peroxidase (GPx)

In the presence of glutathione reductase and NADPH, the oxidation of glutathione (GSH) by cumene hydroperoxide is catalyzed by GPx. Oxidized glutathione (GSSG) is immediately converted into the reduced form with a subsequent oxidation of NADPH to NADP+ (Randox Laboratories, Ltd., Antrim, UK) Decrease in absorbance was measured at 340 nm with UV-spectrophotometer Multiskan ^TM^ Go Microplate (Thermo Scientific ^TM^).

### 2.10. SOD/GPx and GAP

We calculated the SOD/GPx ratio with the values obtained for both enzymes. Regarding GAP, we use the following equation [21]:

GAP = (AT − [(albumin (mmol) × 0.69) + uric acid (mmol)])

### 2.11. Oxidative Stress Score

The oxidative stress score (OSS) was evaluated considering the lipoperoxide (LPO) parameters measured by TBARS, SOD, GPx, TAS, SOD/GPx ratio, and GAP. Alternative cut-off values were defined for each parameter using the 90th percentile of the healthy population, remaining as follows: LPO ≥ 0.340 mmol/L; SOD ≤ 170 IU/mL; GPx ≤ 5500 IU/L; TAS ≤ 0.9 mmol/L; SOD/GPx ≥ 0.023, and GAP ≤ 190 mmol/L. A score of 1 was given to each value above or below the limit and finally the index score was calculated from 1 to 6, which represents the severity of the change in the biomarkers.

### 2.12. Serum 8-Isoprostanes

Measurements were performed with the competitive enzyme-linked immunosorbent assay (EIA) technique using the 8-Isoprostane EIA Kit, Item No. 516351 from Cayman Chemical Company (Ann Arbor, MI, USA). It is based on competition between free 8-isoprostane and conjugated 8-isoprostane-acetylcholinesterase (AChE) (8-isoprostane tracer) for a limited number of binding sites of the 8-isoprostane-specific rabbit antiserum. The concentration of the tracer 8-isoprostane is constant, while the concentration of free 8-isoprostane varies. This rabbit 8-isoprostane complex antiserum (either free or tracer) binds to rabbit monoclonal antibody mouse IgG that has been bound to the well of the plate, then the plate is washed to remove unbound reagents and then an AChE substrate is added to the well where the development of a yellow color is observed as a product of the enzymatic reaction, which was read at 410 nm with a spectrophotometer and the intensity was proportional to the amount of free 8-isoprostanes present in the well. Values were expressed as pg/µL.

### 2.13. 8-Hydroxydeoxyguanosine (8-OHdG)

Measurement of 8-OHdG was carried out with an ELISA kit (Wuhan Fine Biotech Co., Ltd., Wuhan, Hubei, CHN) according to the manufacturer’s instructions. The 8-OHdG monoclonal antibody reacts competitively with plate-bound 8-OHdG and 8-OHdG in serum samples. The addition of the substrate solution resulted in color development in proportion to the amount of anti-8-OHdG antibody bound to the plate. The reaction was stopped with sulfuric acid and the absorbance at 450 nm was measured.

### 2.14. Interleukin Measurement (IL)

Cytokine concentrations were measured using the BD Human Inflammatory Cytokines Cytometric Bead Array (CBA) kit and technique (BD Biosciences, San Jose, CA, USA). Quantifications were performed using the flow cytometric method for measurement and the FCAP ArrayTM software v3.0 for conversion to pg/mL. Aliquots of serum samples were assayed by flow cytometry (CBA Kit, Human Inflammatory Cytokine, BD, San Diego, CA, USA) to determine the levels of IL: IL1-β, IL-6, IL-8, IL-10, and tumor necrosis factor-alpha (TNF-α). For the measurement of CRP, particles coated with anti-human CRP antibodies were used, which were agglutinated by CRP molecules present in the serum samples analyzed. Since the agglutination causes changes in the absorbance proportionally to the concentration of CRP and after comparison with a calibrator, it was possible to determine the exact concentration of the protein. This test was carried out on the Selectra Junior automated equipment (Vital Scientific, Dieren, the Netherlands) under a turbidimetric principle, using a commercial kit from Spinreact (CRP Turbi 1107101L; Girona, Spain).

### 2.15. Sechium edule Formulation

The formulation of the *S. edule* and placebo powder capsules was developed at FES Zaragoza. Later, the treatments (active as placebo) were manufactured and packaged by a pharmaceutical company specialized in the naturopathic field. The *Sechium edule* capsules contained 500 mg of dry powder of *S. edule var. nigrum spinosum.* The fruits were provided by the Interdisciplinary Research Group on *S. edule* in Mexico A.C. (GISeM). 

The analysis of bioactive compounds present in *S. edule* capsules was carried out. The following results were obtained: 154.8 µg cucurbitacin E, 89.9 µg cucurbitacin B, 6.11 µg cucurbitacin D, and 0.71 µg cucurbitacin I; regarding flavonoids, it contains 48.8 µg of naringenin, 14.2 µg of phlorizin, 0.014 µg of apigenin, 45.5 µg of rutin, 2.38 µg of myricetin, and 1.30 µg of quercetin; finally, it contains phenolic acids such as chlorogenic 1.4 µg, protocatechuic 3.3 µg, caffeic 9.3 µg, ferulic 7.0 µg, syringic 8.7 µg, p-coumaric 1.7 µg, gallic 38.8 µg, and p-hydroxybenzoic acid 0.11 µg. The placebo capsules were identical to those of *S. edule*, but contained lactose monohydrate and talc, both pharmaceutical grade and in accordance with the United States Pharmacopeia (USP) (Sigma, St. Louis, MO, USA, EE.UU.).

### 2.16. Statistic Analysis

Data were analyzed using the statistical package IBM SPSS V 21 (Armonk, NY, USA). Descriptive measures and ANOVA of repeated measures were used taking into account the baseline measurement vs. three months and baseline vs. six months of treatment. The chi-square test was used to compare the proportions obtained. Statistical significance was considered at 95%.

## 3. Results

The clinical and anthropometric parameters of the study groups, experimental group (EG) and placebo (GP), before and after treatment at three and six months are shown in Table 1. The EG has a decrease in body weight with limited statistical significance at three and six months (*p* = 0.05), and in the average fat mass at 6 months (*p* = 0.05). Likewise, a decrease in SBP was demonstrated at three and six months with a borderline statistical significance (*p* = 0.05), and in DBP, at three months (*p* = 0.05) and at six months (*p* < 0.05) in the GE.

Regarding the biochemical parameters, the EG showed a statistically significant increase in the concentration of high-density lipoprotein cholesterol (HDL-C) after three months (*p* = 0.05) and six months (*p* < 0.05) of treatment. Furthermore, a decrease in the concentration of HbA1c was found with borderline statistical significance (*p* = 0.05) at six months in the EG (Table 2).

Regarding OxS markers, a statistically significant decrease in LPO concentration was observed at three and six months (*p* < 0.05) in the EG. Additionally, a significant increase in activity and SOD and TAS was found at three and six months (*p* < 0.05), in congruence with a decrease in the OSS in the EG (Table 3). A decrease in the concentration of 8-isoprostanes was also observed with borderline statistical significance (*p* = 0.05) coupled with a statistically significant decrease in 8-OHdG at six months in the EG (*p* < 0.001) (Table 4).

Chronic inflammation markers showed a statistically significant increase in IL-10 at six months (*p* < 0.05) and borderline in IL-6 (*p* = 0.05) in the EG. The other markers did not show significant changes (Table 5).

On the other hand, considering the effect of *S. edule* on the clinical control of MetS, a statistically significant improvement (without MetS) was observed at 3 months (EG, 30% vs. PG, 13%, *p* < 0.05) and at 6 months (EG, 56%, vs., PG, 13%, *p* < 0.05) (Table 6).

## 4. Discussion

MetS is a set of systemic alterations whose pathophysiology is attributed to insulin resistance associated with an excessive flow of fatty acids as well as a pro-oxidative and inflammatory state; it has a high prevalence in the elderly [22]. The fundamental approach to treatment is weight loss through the practice of moderate physical exercise, a diet low in calories and saturated fat, in addition to pharmacological treatment for long periods. However, the results have not been entirely satisfactory. For this reason, some complementary therapeutic alternatives have been proposed such as the consumption of healthy foods, among which fruits and vegetables with a high content of antioxidants and an anti-inflammatory effect stand out [12]. In this regard, some studies have reported that *S. edule* has bioactive compounds with hypoglycemic, anti-inflammatory, antioxidant, hypotensive, and lipogenesis inhibitory effects [7,23].

In the present study, it was observed that the consumption of *S. edule* in patients with MetS induces a decrease in body weight and fat mass six months after treatment with borderline statistical significance, which is consistent with what was reported in a previous study in patients with MetS who consumed 1.5 g of dried fruit of *S. edule* for three months [14]. This finding suggests that the lipolytic effect is maintained in the long term. Likewise, *S. edule* has a high content of insoluble fiber and therefore has a direct effect on the digestive process and elimination of a greater amount of feces [23].

On the other hand, in our study, a decrease in SBP and DBP was observed at three months. This decrease is maintained at six months after treatment, which coincides with that reported in studies in preclinical in vivo models where, after the administration of *S. edule* extracts, a hypotensive and vasodilator effect is observed, due to the high concentration of polyphenols that this fruit contains, which can affect the renin–angiotensin system through the modulation of calcium [24,25]. It has also been reported that this effect can be attributed to the presence of flavonoids as quercetin [14,26].

Regarding the hypoglycemic effect of *S. edule* in this study, a statistically significant decrease in HbA1c was found after six months of treatment, which coincides with what was previously reported by our research group [14], and can be explained by some mechanisms among which we found the increase in insulin release by modifying calcium metabolism in the β cells of the islets of Langerhans, an effect attributed to flavonoids such as quercetin, epicatechin, 2-vicenin, apigenin, and 7-O rutinoside present in high concentrations in *Sechium edule.* Likewise, it has been shown that polyphenols, anthocyanins, and vegetable protein have an inhibitory effect on the expression of protein tyrosine phosphatase 1β (PTP1β), which is a negative regulator of the insulin signaling pathway, whose inhibition translates into an increase in insulin sensitivity, which manifests as a hypoglycemic effect [27,28,29,30,31,32,33].

We also observed a statistically significant increase in HDL-cholesterol in the EG at 3 and 6 months of treatment in contrast to the PG, which coincides with other studies where the same effect is observed [13,14]. This effect may be useful for the prevention and/or control of cardiovascular diseases [31].

Regarding the OxS markers, a statistically significant decrease was found in the serum concentration of LPO and 8-isoprostanes at three and six months in the EG, which implies a decrease in oxidative damage to lipids. This agrees with what was previously reported by our research group [13]. This effect may be due to the high amount of antioxidant molecules present in *S. edule* such as polyphenols (vanillic, gallic, caffeic, and coumaric acids) and flavonoids (naringenin, phloretin, phloridzin, and apigenin) [11]. In this sense, the antioxidant effect of flavonoids has been extensively studied and it has been shown that naringenin, an abundant molecule in *S. edule*, is capable of transferring a hydrogen atom from its OH groups directly to the RL to stabilize the molecule and prevent further damage; likewise, the 5,7-dihydroxy group of ring A increases the stability of the RL by electronic resonance; another mechanism of action of this compound is at the cellular level since it accumulates in the lipid bilayer of the cell membrane, which reduces lipid peroxidation and allows membrane functionality to be maintained [12]. Given the number of bioactive compounds present in *S. edule*, it is possible to assume a synergistic effect that manifests itself with the significant decrease in lipid oxidation markers. In this sense, it is relevant to point out that this antioxidant effect is also observed at the level of cellular DNA since we observed a significant decrease in the concentration of 8-hydroxy-2′-deoxyguanosine, which suggests that the antioxidant components present in *S. edule* are also able to protect at the intracellular level [32].

About the components of the antioxidant system, in the present study, we observed a significant increase in the activity of the SOD enzyme, as well as in the serum total antioxidant capacity and the antioxidant gap (GAP) at three and six months of treatment with *S. edule*, which coincides with previously reported findings and can be explained by the aforementioned bioactive compounds. These results are also consistent with the effect observed on the oxidation of lipids and DNA since we generally observed a decrease in oxidation markers and an increase in the components of the antioxidant system, which is consistent with the statistically significant decrease in the OSS, an indicator that comprehensively evaluates some of the OxS markers [34].

In general, these results suggest that the bioactive compounds present in *S. edule* are capable of modulating the oxidant/antioxidant balance towards a less oxidized state in patients with MetS, which supports the recommendation of consuming *S. edule* concentrate supplements as an adjuvant for the control of OxS in older adults with MetS [33,35].

Regarding inflammation markers, we observed a significant increase in IL-6 and IL-10 in the EG in contrast to the PG. This finding has been previously reported by our research group [11,13]. The observed effect may be due to the ability of flavonoids to block key molecules in inflammatory and prothrombotic processes such as nuclear factor κ-β (NF-κβ), as well as the regulation of polyphenols on the signaling of transcription factors proinflammatory STAT and modulation of mitogen-activated protein kinase (MAPk) and arachidonic acid pathways [36]. Likewise, it has been reported that IL-6 has a modulating effect on the immune system and is attributed to a mediating role in metabolic processes linked to insulin resistance due to increased sensitivity [37]. The increase in IL-6 production shown in this study is relevant since it can contribute to the regulation of glucose and lipid homeostasis and transport [38,39,40]. This is consistent with the hypoglycemic effect and the decrease in fat mass observed in the EG.

Finally, regarding the number of criteria for the diagnosis of MetS, a significant decrease was found in the EG since, both at three and six months, the percentage of patients with MetS was reduced, which coincides with the quantitative reductions observed in the parameters used in the diagnosis. These results agree with what was reported in a previous study [14]. Therefore, these findings support the proposal that the consumption of *S. edule* concentrate could be considered as an adjuvant in the conventional treatment of MetS control or its prevention in older adults. 

## 5. Conclusions

Our findings suggest that the consumption of the *Sechium edule*-based compound has an antioxidant, anti-inflammatory, hypotensive, protective effect against DNA damage, and has a significant effect on the control of metabolic syndrome in older adults.

## Figures and Tables

**Table 1:** Clinical and anthropometric parameters by study group.

Parameter	Placebo Group *n* = 40	Experimental Group *n* = 41	*p*-Value
Age (years)	70.7 ± 7.5	67.2 ± 7.2	0.26
Weight (kg)			
Baseline	78.3 ± 16.4	74.0 ± 11.0	
Three months	77.4 ±15.9	72.6 ± 10.8	0.06
Six months	77.6 ± 13.0	72.0 ± 11.2	0.05
kg of fat mass			
Baseline	39.2 ± 10.6	35.3 ± 8.0	
Three months	37.6 ± 10.8	34.6 ± 7.9	0.22
Six months	38.6 ± 11.1	34.0 ± 7.9	0.05
SMMI (kg/m^2^)			
Baseline	8.37 ± 1.7	8.27 ± 1.4	
Three months	8.30 ± 1.6	8.20 ± 1.6	0.35
Six months	8.24 ± 1.6	8.20 ± 1.6	0.42
Waist circumference (cm)			
Baseline	104.2 ± 14.9	104.0 ± 11.8	
Three months	102.9 ± 13.9	104.9 ± 13.6	0.26
Six months	101.7 ± 14.0	102.9 ± 10.2	0.18
SBP (mmHg)			
Baseline	138.8 ± 10.5	128.9 ± 10.8	
Three months	132.9 ± 12.7	125.4 ± 10.2	0.05
Six months	136.0 ± 12.4	121.6 ± 10.5	0.05
DBP (mmHg)			
Baseline	89.5 ± 7.3	84.1 ± 8.7	
Three months	86.8 ± 7.2	78.2 ± 7.9	0.05
Six months	86.9 ± 7.5	78.6 ± 8.5	0.04

Data are expressed as means ± standard deviation. ANOVA of repeated measures test, significance level 95%. Baseline vs. three months and baseline vs. six months intergroup *p*-value are shown. kg: Kilogram, SMMI: Skeletal muscle mass index. SBP: systolic blood pressure. DBP: diastolic blood pressure.

**Table 2:** Pre- and post-treatment biochemical parameters by study group.

Parameter	Placebo Group *n* = 40	Experimental Group *n* = 41	*p*-Value
Glucose (mg/dL)			
Baseline	113 ± 43	113 ± 37	
Three months	123 ± 43	105 ± 29	0.20
Six months	118 ± 40	103 ± 28	0.14
Cholesterol (mg/dL)			
Baseline	188 ± 39	203 ± 50	
Three months	194 ± 53	206 ± 43	0.46
Six months	183 ± 44	213 ± 41	0.52
Triglycerides (mg/dL)			
Baseline	179 ± 66	178 ± 75	
Three months	169 ± 60	168 ± 58	0.19
Six months	172 ± 67	161 ± 70	0.09
HDL-C (mg/dL)			
Baseline	40.3 ± 10.3	42.6 ± 10.1	
Three months	41.5 ± 8.0	46.0 ± 9.2	0.05
Six months	40.6 ± 10.4	47.4 ± 10.7	0.04
Uric acid (mg/dL)			
Baseline	4.9 ± 1.2	5.0± 1.2	
Three months	5.0 ± 1.3	4.8 ± 1.6	0.08
Six months	4.8 ± 1.2	5.0 ± 1.3	0.10
HbA1c (%)			
Baseline	6.8 ± 1.9	6.3 ± 1.2	
Three months	6.5 ± 1.8	5.6 ± 1.2	0.19
Six months	6.7 ± 1.9	5.9 ± 0.8	0.05

Data are expressed as means ± standard deviation. ANOVA of repeated measures test, significance level 95%. Baseline vs. 3-month and baseline vs. 6-month inter-group *p*-values are shown. HDL-C: High-density lipoproteins. HbA1c: glycosylated hemoglobin.

**Table 3:** Pre- and post-treatment oxidative stress markers by study group.

Parameter	Placebo Group *n* = 40	Experimental Group *n* = 41	*p*-Value
Lipoperoxides (µmol/L)			
Baseline	0.22 ± 0.04	0.30 ± 0.09	
Three months	0.21 ±0.06	0.23 ± 0.08	0.013
Six months	0.21 ±0.03	0.20 ± 0.03	0.011
GPx (U/L)			
Baseline	6896 ± 2235	5759 ± 1838	
Three months	6097 ± 1318	7051 ± 2131	0.16
Six months	4178 ± 1311	6997 ± 3839	0.09
SOD (U/mL)			
Baseline	170 ± 13.3	181 ± 5.4	
Three months	181 ± 12.4	184 ± 8.9	0.01
Six months	162 ± 15.2	183 ± 8.8	0.03
TAS (mmol/L)			
Baseline	1.3 ± 0.23	1.1 ± 0.17	
Three months	1.1 ± 0.21	1.2 ± 0.15	0.04
Six months	1.2 ± 0.15	1.3 ± 0.15	0.015
SOD/GPx			
Baseline	0.0.27 ± 0.009	0.032 ± 0.005	
Three months	0.031 ± 0.007	0.031 ± 0.007	0.21
Six months	0.031 ± 0.011	0.034 ± 0.007	0.64
AOGAP			
Baseline	525 ± 171	356 ± 169	
Three months	270 ± 194	306 ± 222	0.034
Six months	569 ± 213	461 ± 240	0.01
OSS			
Baseline	1.68 ± 0.7	1.76 ± 0.9	
Three months	1.80 ± 1.1	1.59 ± 1.3	0.06
Six months	2.36 ± 1.1	1.45 ± 1.1	0.01

Data are expressed as means ± standard deviation. ANOVA of repeated measures test, significance level 95%. Baseline vs. 3-month and baseline vs. 6-month inter-group *p*-values are shown. SOD: Superoxide dismutase; GPx: glutathione peroxidase; TAS: total antioxidant status; SOD/GPx: SOD/GPx ratio; AOGAP: antioxidant gap; OSS, oxidative stress score.

**Table 4:** Pre- and post-treatment oxidative stress markers by study group.

Parameter	Placebo Group *n* = 40	Experimental Group *n* = 41	*p*-Value
8-isoprostanes (pg/µL)			
Baseline	300.6 ± 23.3	347.7 ± 20.0	
Three months	290.2 ± 24.5	292.4 ± 21.8	0.09
Six months	318.2 ± 18.7	239.3 ± 15.3	0.05
8-OHdG (ng/mL)			
Baseline	31.1 ± 1.6	30 ± 2.1	
Three months	29.8 ± 1.6	24 ± 1.7	0.09
Six months	31.5 ± 1.4	20.5 ± 1.5	0.001

Data are expressed as means ± standard deviation. ANOVA of repeated measures test, significance level 95%. 8-OHdG: 8-hydroxydeoxyguanosine.

**Table 5:** Inflammation markers pre- and post-treatment by study group.

Parameter	Placebo Group *n* = 40	Experimental Group *n* = 41	*p*-Value
IL-12p70 (pg/dL)			
Baseline	4.3 ± 0.3	2.8 ± 0.3	
Three months	4.7 ± 0.4	4.1 ± 0.3	0.18
Six months	4.9 ± 0.3	4.1 ± 0.3	0.20
TNF-α (pg/dL)			
Baseline	4.7 ± 0.4	4.3 ± 0.4	
Three months	4.6 ± 0.3	4.4 ± 0.4	0.19
Six months	4.7 ± 0.3	4.6 ± 0.4	0.22
IL-10 (pg/dL)			
Baseline	3.5 ± 0.3	2.2 ± 0.5	
Three months	1.9 ± 0.2	3.8 ± 0.4	0.05
Six months	2.9 ± 0.4	4.1 ± 0.4	0.04
IL-6 (pg/dL)			
Baseline	8.6 ± 0.4	6.4 ± 0.9	
Three months	6.1 ± 0.4	7.7 ± 0.6	0.09
Six months	8.2 ± 0.4	11.1 ± 0.6	0.05
IL-1β (pg/dL)			
Baseline	9.9 ± 0.5	8.6 ± 0.4	
Three months	10.3 ± 0.3	9.6 ± 0.4	0.76
six months	11.4 ± 0.3	10.2 ± 0.5	0.46
IL-8 (pg/dL)			
Baseline	14.2 ± 0.3	13.5 ± 0.4	
Three months	18.1 ± 0.4	14.8 ± 0.5	0.8
Six months	18.9 ± 0.4	16.5 ± 0.4	0.59
PCR (mg/dL)			
Baseline	0.52 ± 0.08	0.60 ± 0.09	
Three months	0.52 ± 0.12	0.53 ± 0.01	0.5
Six months	0.46 ± 0.08	0.52 ± 0.05	0.5

Data are expressed as means ± standard deviation. ANOVA of repeated measures test, significance level 95%. IL: Interleukin; TNF- α: Tumor necrosis factor α; CRP: C-reactive protein.

**Table 6:** Metabolic syndrome control after of three and six months of treatment.

	Placebo Group *n* = 40		Experimental Group *n* = 41	
	With MetS	Without MetS	With MetS	Without MetS
Baseline	40 (100)	0 (0)	41 (100)	0 (0)
Three months	35 (87)	5 (13)	29 (70)	12 (30) *
Six months	35 (87)	5(13	18 (44)	23 (56) *

Proportions, Chi-square test, significance at 95%, * *p* < 0.05. ≥3 Criteria = presence of MetS; <3 criteria = absence of MetS.

## Data Availability

The data presented in this study are available on request from the corresponding author.
